# Relationship between Fecal Content of Fatty Acids and Cyclooxygenase mRNA Expression and Fatty Acid Composition in Duodenal Biopsies, Serum Lipoproteins, and Dietary Fat in Colectomized Familial Adenomatous Polyposis Patients

**DOI:** 10.1155/2010/862569

**Published:** 2010-10-28

**Authors:** K. Almendingen, A. T. Høstmark, L. N. Larsen, O. Fausa, J. Bratlie, L. Aabakken

**Affiliations:** ^1^Research Centre, Akershus University Hospital, 1478 Lørenskog, Norway; ^2^Faculty of Health, Nutrition and Management, Akershus University College, P.O. Box 423, 2001 Lillestrøm, Norway; ^3^Institute of Chemistry, Biotechnology and Food Science, Norwegian University of Life Sciences, 1432 Aas, Norway; ^4^Section of Preventive Medicine and Epidemiology, University of Oslo, P.O. Box 1130, Blindern, 0318 Oslo, Norway; ^5^EpiGen Institute, Research Centre, Akershus University Hospital, 1478 Lørenskog, Norway; ^6^Department of Gastroenterology, Rikshospitalet University Hospital, 0027 Oslo, Norway

## Abstract

A few familial adenomatous polyposis studies have focused upon faecal sterols and bile acids but none has analysed the fecal content of fatty acids. We report here findings of an observational study on 29 colectomized familial adenomatous polyposis patients that describe the fecal content of fatty acids, and relate this to the proportions of fatty acids and levels of cyclooxygenase mRNA expression in duodenal biopsies, levels of serum lipoproteins, and diet. In the ileostomy group separately (*n* = 12), the fecal content of arachidonic acid was correlated negatively to the proportions of eicosapentaenoic acid and docosahexaenoic acid in duodenal biopsies. Total serum-cholesterol was negatively correlated to the fecal content of saturates and monounsaturates. The fecal palmitoleic acid/palmitic acid ratio was positively correlated to the levels of cyclooxygease-2 expression in duodenal biopsies.In the ileal-pouch-anal anastomosis group separately (*n* = 17), significant correlations were found between the fecal contents of oleic acid, linoleic acid, and alpha-linolenic acid, and the proportions of myristic acid, oleic acid and eicosaenoic acid in duodenal biopsies. Dietary monounsaturates were positively correlated to different fecal fatty acids. Future studies should focus on molecular mechanisms relevant to fatty acid metabolism, inflammation, and angiogenesis, in addition to nutrition.

## 1. Introduction

Familial adenomatous polyposis [1] account for 1% of colorectal cancers, and provides a model of APC inactivation as an early genetic event for the approximately 80%–85% of cancers that develop from sporadic polyps. Colorectal cancers arising in patients with familial adenomatous polyposis can be largely prevented by polyp surveillance and prophylactic colectomy [2]. Total proctocolectomy with construction of a conventional ileostomy or ileoanal anastomosis with preparation of an ileal pouch, has various effects on the function of the terminal ileum and the intestinal bacterial flora [3]. This may deteriorate cholesterol metabolism, as absorption of cholesterol in duodenum and jejunum requires micellar solubilization with bile acids, fatty acids, monoglycerides, and phospholipids [3]. Hypothetically, ileal-pouch-anal anastomosis and ileostomy patients might differ with regard to the presence of various fatty acids in feces and their relationship to other reflections of lipoprotein metabolism, but we found no previous study focusing upon this issue.

Dietary fatty acids are incorporated into blood and tissues, and the fatty acid composition in these tissues are often used as biomarkers of fat intake. Furthermore, the fecal amount and composition of fatty acids reflect fat ingestion, intestinal fatty acid absorption, and the activity of colonic bacteria [4]. Although some of the discrepancies between studies may be due to the use of different methods to analyze fatty acids, differences in diet, or the fact that assessments have been performed in different body compartments, modifications in the metabolism of fatty acids have been suggested in cancer patients [5, 6]. It is not clear at what steps in the multistage carcinogenesis process a possible distorted fatty acid metabolism occurs. Notably, if such alterations occur in the development of carcinogenesis, this may affect the biological functions of essential fatty acids and their derivates [5].

Very little is known about fatty acid metabolism in familial adenomatous polyposis, although chemoprevention affecting the fatty acid derivates and the cyclooxygenase enzymes is often administered to familial adenomatous polyposis patients. Deregulation of the cyclooxygenase-2 pathway appears to affect tumorigenesis via a number of distinct mechanisms: promoting tumour maintenance and progression, encouraging metastatic spread, and perhaps even participating in tumour initiation [7]. Cyclooxygenase-1 and -2 are the rate limiting enzymes in the synthesis of prostaglandins and thromboxanes [8]. Arachidonic acid is the main substrate for these enzymes, leading to the synthesis of prostaglandins which have growth promoting effects. Substituting arachidonic acid with omega-3 fatty acids has been shown to lead to the production of less potent prostaglandins [9]. Since cyclooxygenase-2 is a fatty acid metabolising enzyme, the relationships between cyclooxygenase-2 and fatty acid composition of different tissues is of interest. Colectomized familial adenomatous polyposis patients had a deviant fatty acid profile with high levels of arachidonic acid and docosahexaenoic acid and low levels of linoleic acid and alpha-linolenic acid in serum phospholipids, which is in accordance with studies in patients with other types of cancers [5, 10–13]. In a previous familial adenomatous polyposis study [14], comparable treatment effects of a cyclooxygenase-2 inhibitor were observed on the fatty acid composition in serum phospholipids and duodenal lesions, presumably and most importantly the nonbeneficial effects involving essential fatty acids. 

We report here findings from an observational study in colectomized familial adenomatous polyposis patients. The results describe the content of fatty acids in feces, and relate this to the proportions of fatty acids and levels of cyclooxygenase mRNA expression in duodenal biopsies, diet, and levels of serum lipoproteins. Because the effects of ileostomy construction may differ from that of ileal-pouch-anal anastomosis because of scarcity of the bacterial flora and different surface area of the terminal ileum [3], we did separate analyses for these two groups. Previous familial adenomatous polyposis studies have focused upon faecal sterols and bile acids [15–17], but none has to the best of our knowledge analysed the content of fatty acids in feces, including very long chained fatty acids.

## 2. Material and Methods

### 2.1. Patients

Data from the present study are taken from a randomized double-blind placebo-controlled intervention study with a cyclooxygenase-2 inhibitor [12, 14, 18]. The main aim was to compare the effect of Rofecoxib treatment on duodenal lesions (data in preparation). The present study comprises data from baseline for only the patients operated with ileal-pouch-anal anastomosis and ileostomy (77% of total study group). Patients were recruited from a Norwegian familial adenomatous polyposis registry. All of them had been colectomized, and duodenal lesions had been verified by endoscopy and histology. Biopsies were taken partly from macroscopically normal mucosa, partly from representative visible adenoma. Rikshospitalet University Hospital is a highly specialized university hospital with national responsibilities in the area of complicated treatments, such as follow-ups on the Norwegian familial adenomatous polyposis patients. Inclusion criteria were verified familial adenomatous polyposis, colectomy, 18–70 years of age, and documented duodenal lesions graded as Spigelman I, II, or III and the largest adenoma∖10 mm. Exclusion criteria were indications for surgical treatment, suspected or documented intestinal obstruction or stenosis, patients unwilling or unable to adhere to protocol, known cardiac failure requiring medical treatment, and pregnancy. All patients were on a free diet. As for the adenomas sampled, they were all assessed histologically as mild/moderate dysplasia, all below 10 mm and all flat, located in the descending part of the duodenum. No foci of cancer were found in any of the lesions.

### 2.2. Determinations

Samples of feces were transferred to tubes and mixed to make a homogeneous mass of which 100–200 uL were used for lipid extraction by adding 50 uL butanol, 100 *μ*L H_2_O, 1 mL methanol, and 1 mL chloroform. After 10 min under N_2_, the mixture was filtered and the filter washed with 1 ml chloroform + 1 mL chloroform/methanol (2 : 1) + 0.8 mL 0.73% NaCl. After mixing for 15 s, the tubes were centrifuged for 30 min at 1000 g. The upper phase was discarded and the lower phase transferred to new tubes and evaporated under N_2_ at 65°C using a heat block. Then 0.4 mL 0.5 N NaOH in methanol was added and the solution kept for 7 min at 100°C under nitrogen_. _After cooling to room temperature, 0.5 mL 12% BF_3_ was added and the solution kept for 10 min at 100°C under nitrogen. After cooling to room temperature, 1 mL heptan was added under nitrogen, and mixing for 30 s, 1 mL of saturated NaCl solution was added. After mixing, the solution was centrifuged for 10 min at 1000 g, and the upper phase transferred to new tubes. The extraction procedure was performed one more time. Finally, the heptan was evaporated and 300 *μ*L hexan added before continuing with the gas chromatography procedure referred to earlier [12].

### 2.3. Habitual Diet

Dietary intake was assessed by a validated food frequency questionnaire, designed to cover as much of the total habitual diet as possible. Questions were related to habitual frequency of consumption and the amount of foods eaten during the last year. They were asked to identify their habitual choices of edible fats by pointing at specially prepared pictures, in order to increase the validity of the estimate of dietary fat [18].

### 2.4. RNA Isolation from Duodenal Lesions and Real-Time Polymerase Chain Reaction

Lesions were taken and stored in RNAlater solution previous to freezing at −80°C. Each biopsy was put into a tube containing 600 *μ*L Trizol and immediately mixed on a MixerMill (Retsch GmbH, Germany). RNA was isolated from the samples after standard methods. Gene expression analyses were carried out on a 7900 HT real-time PCR machine from Applied Biosystems. Based on the results from running a housekeeping gene test (“TaqMan Human Endogenous Control Plate,” Applied Biosystems) with RNA from isolated human leucocytes (data not shown). Tata binding protein (TBP) was chosen as housekeeping gene. The primers and probes were initially designed as three assays per gene and validated for efficiency and specificity. The best of the three was then chosen. The primers and probes for the COX-1 assay were: forward primer: 5′-CTTCCAGGAGCTCGTAGGA-3′, probe: 5′-AGAAGGAGATGGCAGCAGAGTTGGAG-3′, and reverse primer: 5′-ACGCATCAATGTCTCCATACAAT-3′. COX-2 forward primer: 5′-TGGAACATGGAATTACCCAGT-3′, probe: 5′-TGTTGAATCATTCACCAGGCAAATTGCT-3′, and reverse primer: 5′-TCCTACCACCAGCAACCCT-3′. GUS forward primer: 5′-GAAAATATGTGGTTGGAGAGCTCATT-3′, probe: 5′-CCAGCACTCTCGTCGGTGACTGTTCA-3′, and reverse primer: 5′-CCGAGTGAAGATCCCCTTTTTA-3′. TBP forward primer: 5′-CTGGAAAAGTTGTATTAACAGGTGC-3′, probe: 5′-AGCAGAAATTTATGAAGCATTTGAAAACATCTACCCTATT-3′, and reverse primer: 5′-CATTACGTCGTCTTCCTGAATC-3′. 

TaqMan Universal PCR Master Mix (Applied Biosystems) was added as reaction mix. The reaction conditions were initiated by a step of 2 min. at 50°C and 10 min. at 95°C, followed by 40 cycles of denaturation at 95°C for 15 sec. and annealing at 60°C for 1 min. Standards and samples was analyzed in triplicates for all assays. A combination of cDNA from several samples were made and diluted in order to make a dilution curve that was included on each plate. The average of the three values for each gene was divided by the average of the corresponding TBP values, generating a normalized value of the gene expression which is a unit less value used to compare the relative amount of mRNA for each gene in the different samples.

### 2.5. Ethics Approval

The study was performed in accordance with the Helsinki Declaration. Patients were informed by a physician, and in addition thoroughly written information was given to all patients. The protocol was explained to the subjects that had to give their consent before inclusion. No honorarium was offered. Retrospective registration was done on 02/13/09 at ClinicalTrials.gov. The study protocol (RH01/01) was approved by the Norwegian health authorities and the Regional Committee of Medical Ethics 20/06/2002 (reference: S-02127).

### 2.6. Data Analysis

Nonparametrical statistical methods were chosen, as some of the variables were skewed and the number of observations limited. Median values with quartiles are presented unless otherwise stated. Relations between variables were investigated by using Spearman's correlation coefficients (rho). All statistical analyses were performed with the SPSS 12.0 and Excel software for Windows.

## 3. Results

### 3.1. Background Characteristics

Background characteristics of the patients are given in Table 1. Age and fecal content of palmitolic acid, linoleic acid, and arachidonic acid differed between the ileostomy and the ileal-pouch-anal anastomosis patients (Table 1).

### 3.2. The Ileostomy Group

The content of linoleic acid in feces correlated negatively to the proportion of eicosaenoic acid in duodenal biopsies (*r* = −0.6, *P* < .05). The content of arachidonic acid in feces was correlated negatively to the proportions of eicosapentaenoic acid (*r* = −0.7, *P* = .02) and docosahexaenoic acid (*r* = −0.7, *P* = .008) in duodenal biopsies. Total cholesterol were negatively correlated to the content of palmitic acid (*r* = −0.7, *P* = .03), palmitoleic acid (*r* = −0.7, *P* = .02), stearic acid (*r* = −0.7, *P* = .02), oleic acid (*r* = −0.7, *P* = .02), linoleic acid (*r* = −0.8, *P* = .002), and monounsaturated fatty acids (*r* = −0.7, *P* = .02) in feces. The levels of triglycerides was negatively correlated to the content of linoleic acid (*r* = −0.8, *P* = .003) and eicosaenoic acid (*r* = −0.7, *P* = .03) in feces. No significant correlations were found between the content of different fatty acids in feces and the fatty acid dietary intake (data not shown). 

**Figure 1:**
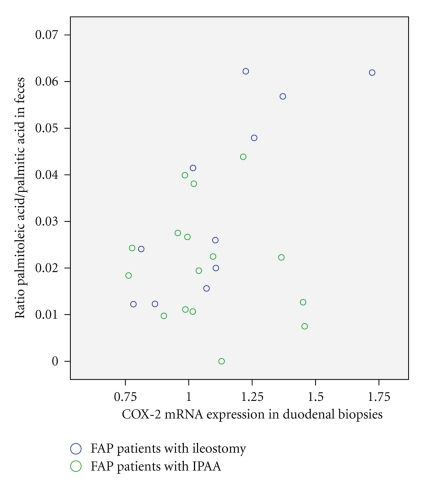
The scatterplot shows the ratios of palmitoleic acid/palmitic acid in feces samples and the levels of cycooxygenase-2 expression in duodenal biopsies (ileostomy group, *n* = 12: *r* = 0.8, *P* = .003; ileal-pouch-anal anastomosis group, *n* = 16: *r* = − 0.2, *P* = .5).

With the exception of the palmitoleic acid/palmitic acid ratio and levels of cyclooxygenase-2 expression in duodenal biopsies (*r* = 0.8, *P* = .003), no relationships were found between fatty acids in feces and the levels of cyclooxygenase mRNA expression (Figure 1).

### 3.3. The Ileal-Pouch-Anal Anastomosis Group

Significant correlations were found between the content of oleic acid in feces and the proportion of myristic acid (*r* = 0.8, *P* < .001), oleic acid (*r* = 0.5, *P* < .05), and eicosaenoic acid (*r* = −0.5, *P* = .03) in duodenal biopsies. Moreover, the content of linoleic acid in feces correlated significantly to the proportion of myristic acid (*r* = 0.8, *P* < .001) in duodenal biopsies, whereas the content of alpha-linolenic acid in feces correlated inversely to eicosaenoic acid (*r* = −0.6, *P* < .05) in duodenal biopsies. The content of palmitic acid in feces was positively correlated to levels of LDL-cholesterol (*r* = 0.05, *P* = .05). No significant correlations were found between the content of different fatty acids in feces and the levels of cyclooxygenase mRNA expression in duodenal biopsies (data not shown). 

Several different fatty acids in feces correlated positively to dietary intake of fatty acids, most predominantly monounsaturated fatty acids (Table 2).

## 4. Discussion

As expected, we found a difference between ileal-pouch-anal anastomosis and ileostomy patients concerning associations between fecal fatty acid composition and other variables involved in lipoprotein metabolism. Possibly, our results relate to metabolic differences caused by the different intestinal reconstructions, but the data are not suitable to explain the findings.

We found no significant relationships between the levels of cyclooxygenase mRNA expression in duodenal biopsies and the content of fatty acids in feces, except for the estimates of the content of delta-9-desaturase (stearoyl-CoA desaturase, SCD) activity, namely the ratio of palmitolic acid/palmitic acid [19, 20] in the ileostomy group. Stearoyl-CoA desaturase is the central lipogenic enzyme catalyzing in vivo reactions in the synthesis of monounsaturated fatty acids, particularly oleic acid and palmitoleic acid, which are the major monounsaturated fatty acids of membrane phospholipids, triglycerides, wax esters, and cholesteryl esters. Both delta-9-desaurase and cyclooxygenase-2 are PPAR alpha regulated, although this possible link is only a speculation. A study in healthy subjects showed that human fecal water contains components can affect both the cyclooxygenase-2 protein level and enzymatic activity [21]. One recent study claims that it is possible to detect cyclooxygenase-2 mRNA in feces of colorectal cancer patients irrespective of clinical stage [22]. Notably, increased cyclooxygenase-2 expression in duodenal compared with colonic tissues in familial adenomatous polyposis was recently reported [23]. 

The significant negative correlation between content of arachidonic acid in the feces and amounts of omega-3 fatty acids in duodenal biopsies which was found in the ileostomy group is interesting [24], since substitution of arachidonic acid with omega-3 fatty acids has been shown to lead to the production of less potent prostaglandins [9]. A previous familial adenomatous polyposis study established a positive correlation between the reduction in tissue prostanoid levels and clinical response, as measured by the reduction in size and number of adenomas, when patients were treated with sulindac [25]. However, this correlation only became consistently significant when prostanoids were assayed after tissue homogenates were first incubated with arachidonic acid [25]. We may suspect transformation of linoleic acid to arachidonic acid in feces, but data are limited as with regard to feces from colectomized familial adenomatous polyposis subjects. The main dietary-source of arachidonic acid is animal products. However, the dietary assessment showed that the intake of such foods was normal among these familial adenomatous polyposis patients [18]. 

Dietary controlled intervention studies on ileostomy subjects have shown that dietary manipulation with fat and fiber may modify cholesterol absorption and sterol excretion [4]. In the ileostomy group, levels of total cholesterol were negatively correlated to the content of palmitic acid, palmitolic acid, stearic acid, oleic acid, linoleic acid, and monounsaturated fatty acids (<18 C atoms) in feces, which is interesting since the present levels of total cholesterol are lower than those among a healthy reference group [18]. In the ileal-pouch-anal anastomosis group separately, the content of monounsaturated fatty acid in feces was positively correlated to dietary intake of monounsaturated fatty acids in particular. Moreover, positive correlations were found between the content of oleic acid in feces and the proportion of myristic acid, oleic acid, and eicosaenoic acid in duodenal biopsies. The precise role of monounsaturated fatty acids synthesis in cell proliferation and programmed cell death remains unknown. The strong correlation of high levels of monounsaturated fatty acids and neoplastic phenotype may suggest that the regulation of stearoyl-CoA desaturase must play a role in cancer development. 

This was an observational study, and not a metabolic balance study that was designed to investigate the metabolism of fatty acids in familial adenomatous polyposis patients. Although some studies have found that the absorption rate of fatty acids are unaffected by age [26, 27], we cannot exclude the possibility that some of the observed differences between the ileal-pouch-anal anastomosis and ileostomy patients might be due to age, rather than type of surgery, since lipoprotein clearance has been shown to be altered by age [28], and that might affect the relationship between dietary fatty acids and the fatty acid profiles found in tissues. Moreover, no conclusions can be drawn from the present study because this is an observational study. The subgroup analyses were not designed a priori to detect these differences. However, the study addresses the need to learn more about the dietary and lifestyle habits of this subset of at-risk patients. The present study includes a number of patients close to the maximum possible number of eligible Norwegian familial adenomatous polyposis patients [12]. We may, however, suspect that the low sample size and use of a food frequency questionnaire [14], may have weakened any associations between the content of fatty acids in feces and dietary intake. Notably, these data do not contradict that the nutrient intake among these patients should at least meet the recommendations for healthy subjects [18]. Nevertheless, we suggest that these data are compelling enough to suggest that future familial adenomatous polyposis studies should investigate overall fatty acid metabolism, molecular mechanisms relevant to fatty acid metabolism, inflammation, and angiogenesis, in addition to nutrition requirements.

In conclusion, we found a difference between ileal-pouch-anal anastomosis and ileostomy patients concerning associations between fecal fatty acid composition and other variables involved in lipoprotein metabolism. We may suggest that fatty acid content in feces is related to dietary intake, serum lipids, and fatty acid composition in duodenal biopsies, even in colectomized familial adenomatous polyposis patients. This observational study may represent hypothesis-generating suggestions for future familial adenomatous polyposis studies.

## Figures and Tables

**Table 1:** Baseline characteristics and fecal composition of fatty acids in colectomized patients with familial adenomatous polyposis. Results are presented as median and 25 and 75 percentiles.

Variables	Ileostomy (*n* = 12)	IPAA (*n* = 17)	*P* value
Age, year	51 (36, 70)	36 (24, 61)	.001
Age at colectomy, year	28 (19, 48)	21 (10, 45)	.07
Body mass index, kg/m^2^	26.6 (19.8, 35.2)	25.5 (17.2, 30.8)	.4
Dietary total fat (% of energy)	31 (27, 37)	30 (29, 36)	.8
SAT	13 (11, 14)	11 (10, 13)	.3
MUFA	10 (9, 12)	10 (9, 11)	.5
PUFA	6 (5, 9)	7 (6, 9)	.3
Total-cholesterol (mmol/L)	4.9 (4.2, 6.0)	4.7 (3.9, 5.6)	.5
HDL-cholesterol (mmol/L)	1.8 (1.4, 2.1)	1.5 (1.3, 1.8)	.1
LDL-cholesterol (mmol/L)	2.8 (2.4, 3.8)	3.3 (2.1, 3.9)	.9
Triglycerides (mmol/L)	1.2 (0.9, 1.5)	1.0 (0.6, 1.5)	.3
*Fatty acids in feces (weight %): *			
Myristic acid	1.4 (0.9, 3.9)	1.1 (0.6, 1.4)	.1
Palmitic acid	29.7 (26.1, 36.3)	32.7 (19.4, 38.4)	.7
Palmitolic acid	1.1 (0.5, 1.5)	0.5 (0.3, 0.7)	.003
Stearic acid	21.2 (13.8, 25.8)	28.1 (14.6, 36.5)	.2
Oleic acid	18.9 (16.6, 31.4)	17.7 (12.6, 37.9)	.9
Linoleic acid	16.7 (13.0, 25.3)	14.7 (10.7, 23.0)	.4
Alpha-linolenic acid	1.6 (0.6, 2.8)	0.7 (0.2, 1.3)	.04
Arachidonic acid	0.9 (0.4, 1.5)	0.2 (0.1, 0.8)	.04
*COX mRNA expression in duodenal biopsies*			
COX-1	0.86 (0.74, 0.95)	0.92 (0.83, 1.00)	.1
COX- 2	1.11 (0.9, 1.25)	1.02 (0.96, 1.22)	.6

The values for eicosenoic acid and eicosadienoic acid are not shown, since these values were close to zero.

COX: cyclooxygenase; IPAA: ileal-pouch-anal anastomosis; MUFA: monounsaturated fatty acids; PUFA: polyunsaturated fatty acids; SAT: saturated fatty acids.

**Table 2:** Spearman correlation coefficients (*r*) between fatty acid composition of feces and dietary fatty acids in colectomized familial adenomatous polyposis patients operated with ileal-pouch-anal anastomosis.^1^

Fecal fatty acids						
Dietary fatty acids	Palmitic acid	Palmitoleic acid	Oleic acid	EPA	DHA	Sum MUFAs
Oleic acid	0.5(*)	0.6(*)	0.6	0.2	0.2	0.5(*)
Linoleic acid	0.5	0.5(*)	0.5(*)	0.3	0.3	0.5(*)
Arachidonic acid	0.3	0.2	0.5	0.6(*)	0.5(*)	0.5
Sum MUFAs	0.5(*)	0.6(*)	0.4	0.2	0.2	0.5(*)

*Correlation is significant at the 0.05 level (2-tailed).

^1^No significant coefficients of correlation were seen for the ileostomy group.

The values for eicosenoic acid and eicosadienoic acid are not shown, since these values were close to zero.

DHA: docosahexaenoic acid; EPA: eicosapentaenoic acid; MUFA: monounsaturated fatty acids.
